# Supplementary material for: Identification and New Indication of Melanin-Concentrating Hormone Receptor 1 (MCHR1) Antagonist Derived from Machine Learning and Transcriptome-Based Drug Repositioning Approaches
Source: Int J Mol Sci. 2022 Mar 30;23(7):3807. doi: 10.3390/ijms23073807 (PMC8998904; doi:10.3390/ijms23073807)
Supplement: Supplementary file 1 [file ijms-23-03807-s001.zip › ijms-1642592-supplementary.pdf]

Supplementary Information

Identification and new indication of melanin-concentrating hormone receptor 1 (MCHR1) antagonist derived from machine learning and transcriptome-based drug repurposing approaches

Gyutae Lim [1], Ka Young You [1,5], Jeong Hyun Lee [1,5], Moon Kook Jeon [1], Byung Ho Lee [1], Jae Yong Ryu [3,4,*], and Kwang-Seok Oh [1,2,*]

[1]Data Convergence Drug Research Center, Korea Research Institute of Chemical Technology, 141 Gajeong-ro, Yuseong-gu, Daejeon 34114, Republic of Korea; gyutae@krict.re.kr (G.L.); uuuky@krict.re.kr (K.Y.Y.); jhlee@krict.re.kr (J.H.L.); moteta@krict.re.kr (M.K.J.); bhlee@krict.re.kr (B.H.L.); ksoh@krict.re.kr (K.S.O.)

[2]Department of Medicinal and Pharmaceutical Chemistry, University of Science and Technology, 217 Gajeong-ro, Yuseong-gu, Daejeon 34113, Republic of Korea

[3]Department of Biotechnology, Duksung Women's University, 33 Samyang-ro 144-gil, Dobong-gu, Seoul 01369, Republic of Korea; jyryu@duksung.ac.kr (J.Y.R.)

[4]Institute of BIO-ICT convergence technology, Duksung Women's University, 33 Samyang-ro 144-gil, Dobong-gu, Seoul 01369, Republic of Korea

[5]Graduate School of New Drug Discovery and Development, Chungnam National University, Daejeon 34134, Republic of Korea

*Corresponding author

Jae Yong Ryu, Department of Biotechnology, Duksung Women's University, 33 Samyang-ro 144-gil, Dobong-gu, Seoul 01369, Republic of Korea

Tel: +82-2-901-8414; E-mail: jyryu@duksung.ac.kr

Kwang-Seok Oh, Data Convergence Drug Research Center, Korea Research Institute of Chemical Technology

141 Gajeong-ro, Yuseong-gu, Daejeon 34114, Republic of Korea.

Tel: +82-42-860-7547; Fax: +82-42-861-4246; E-mail: ksoh@krict.re.kr

Supplementary Figures

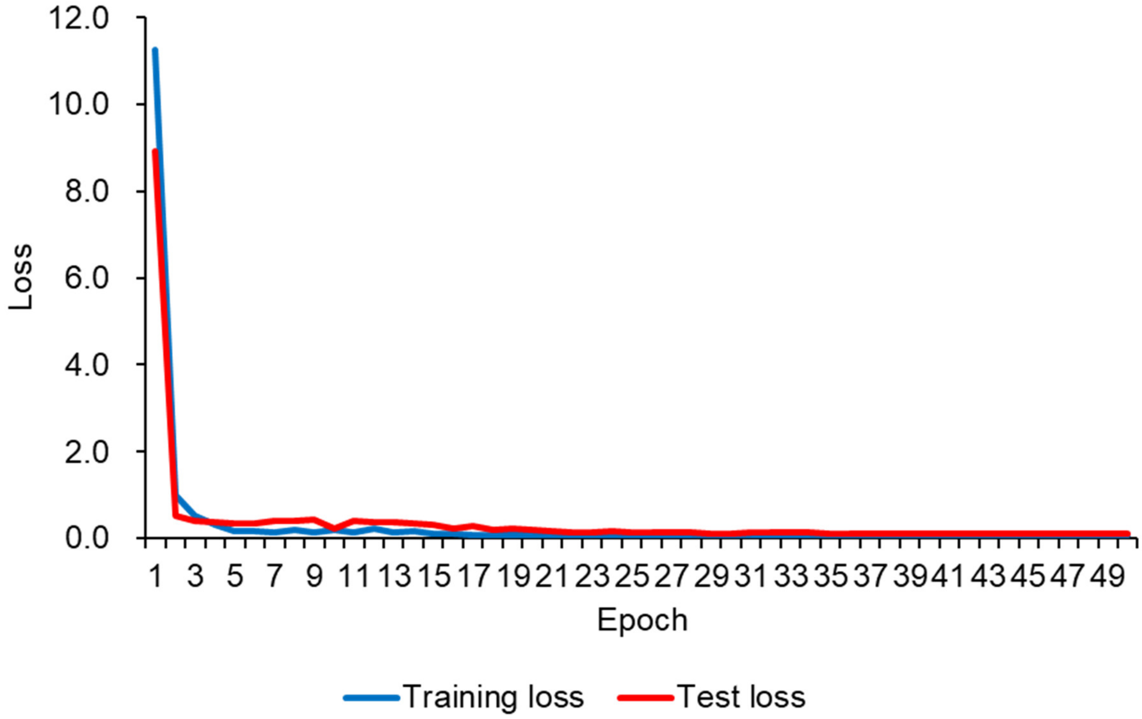

Figure S1. Loss curves of descriptor-based DNN (deep neural network) for 50 epochs. Loss value in each epoch was obtained from the final training procedure after optimization of the descriptor-based DNN architecture.

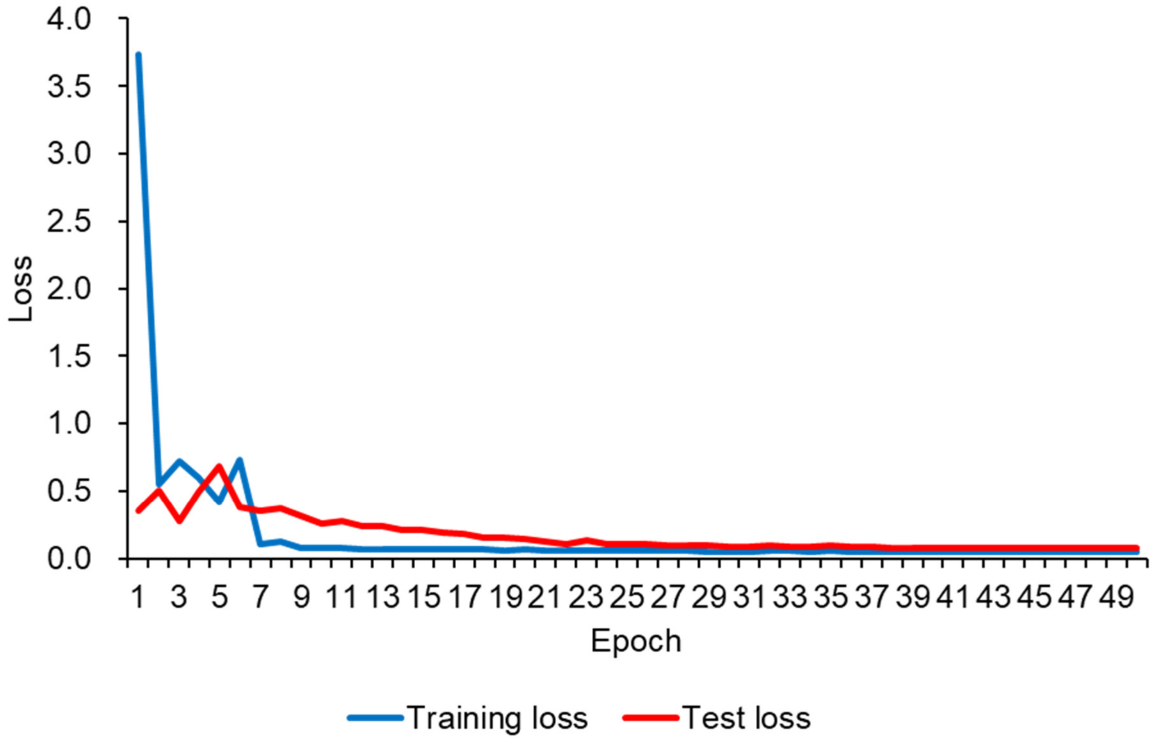

Figure S2. Loss curves of fingerprint-based DNN for 50 epochs. Loss value in each epoch was obtained from the final training procedure after optimization of the fingerprint-based DNN architecture.

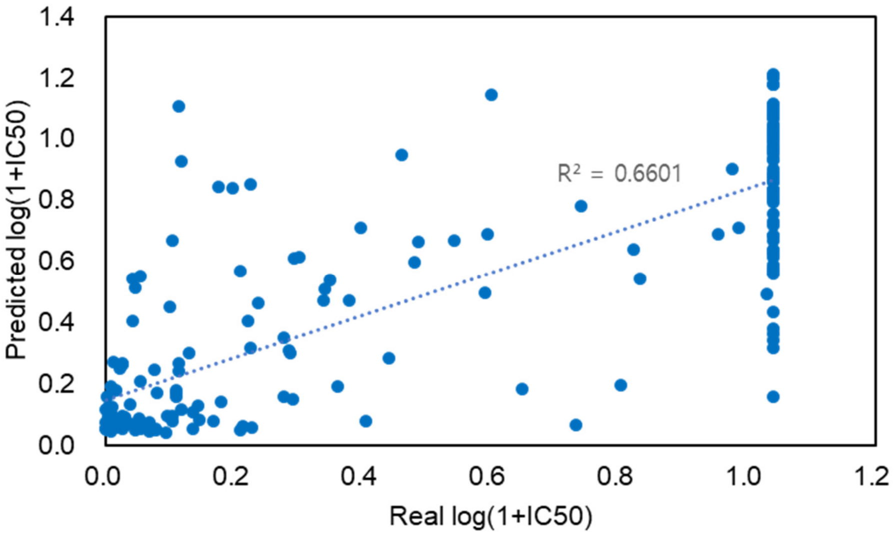

Figure S3. Comparison of real and predicted IC_{50} values on test dataset predicted with descriptor-based DNN.

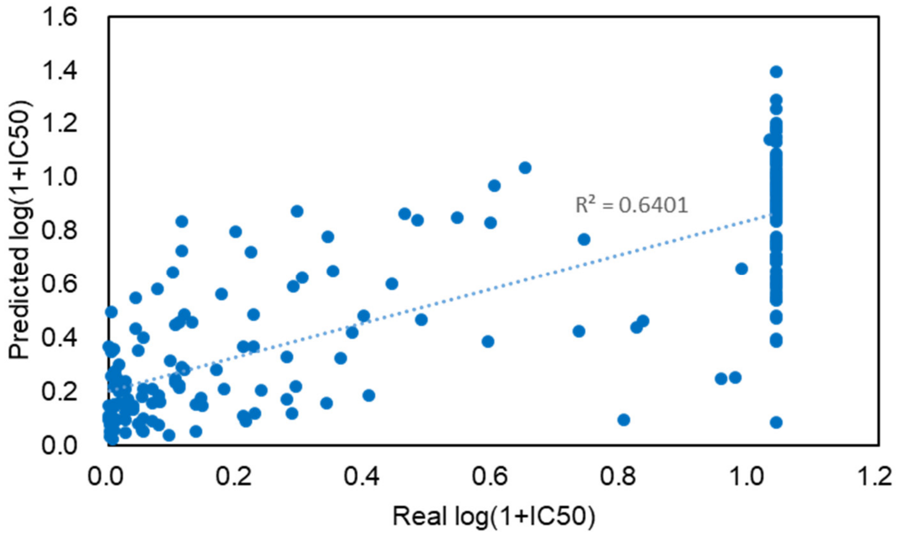

Figure S4. Comparison of real and predicted IC_{50} values on test dataset predicted with fingerprint-based DNN.

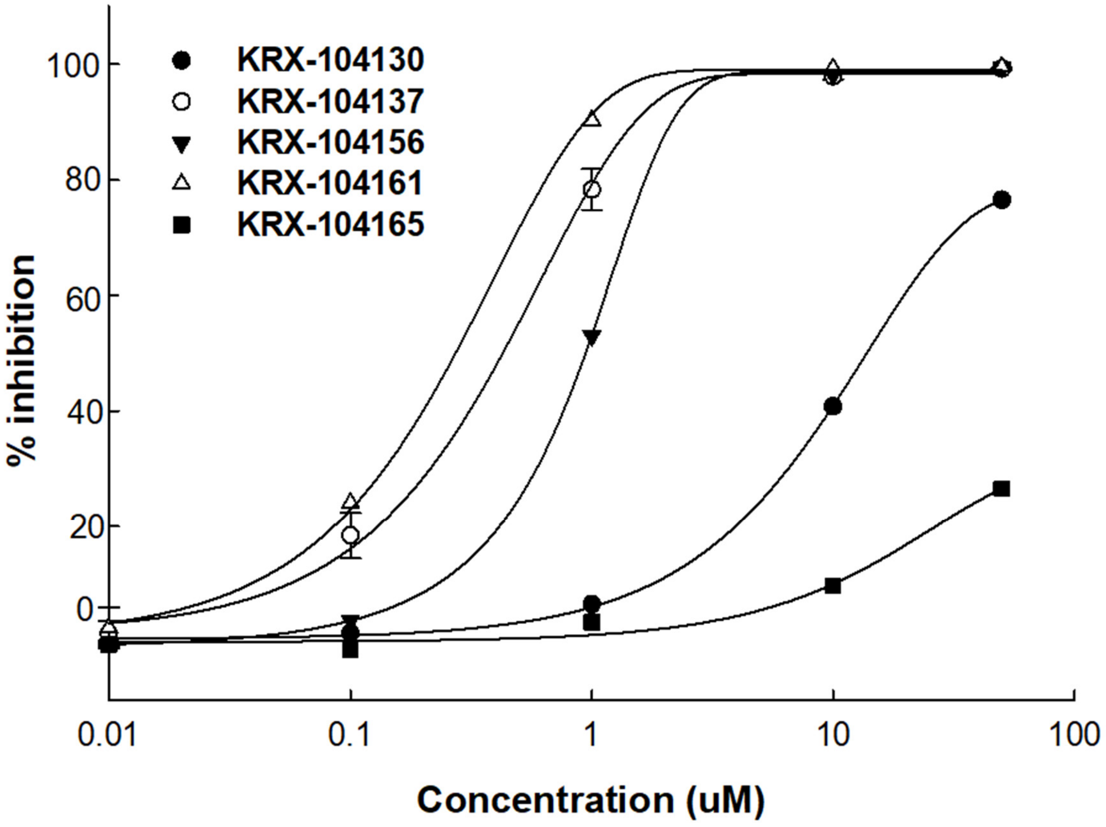

Figure S5. Results of hERG patch clamp activity of compounds.

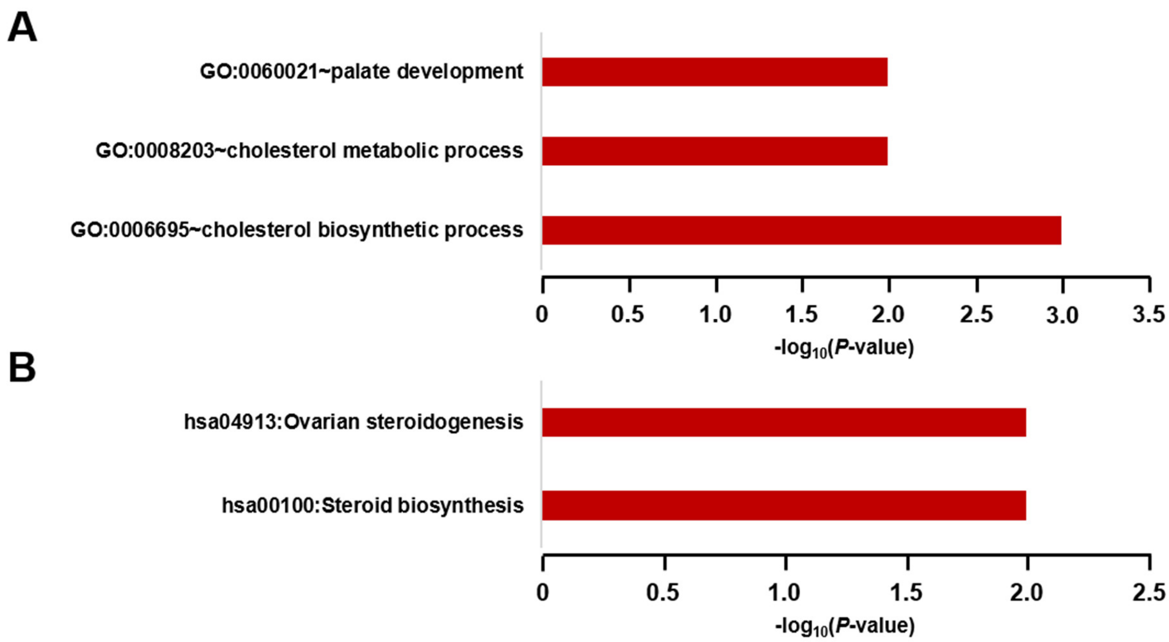

Figure S6. Results of (A) GO and (B) KEGG pathway enrichment analysis of nine common expression genes.

Table S1. Hyperparameters considered in this study

Hyperparameter	Considered Values
Dropout	[1e-4, 1e-1]
Optimizer	{adam, sgd}
Learning rate	[1e-4, 1e-1]
L2 regularization rate	[1e-5, 1e-2]
Batch size	{64, 128, 256}
Number of hidden layers for DNN	[1, 6]
Number of hidden nodes for DNN	[128, 512]

Table S2. Model performance according to the feature number.

Number of features	MSE (descriptor-based DNN)	MSE (fingerprint-based DNN)
4	0.107419911	0.21144493
6	0.097933789	0.215005423
8	0.095514624	0.211168945
10	0.096400381	0.213130072
12	0.087199683	0.213911336
14	0.08989341	0.215021391
16	0.101556298	0.209222856
18	0.089999748	0.213104152
20	0.10280343	0.21506535
22	0.09572569	0.209033838
24	0.09986852	0.203798517
26	0.086370617	0.217191686
28	0.098629042	0.210982217
30	0.086209713	0.209720522
32	0.090939849	0.126496404
34	0.096478134	0.124006814
36	0.104143513	0.117156515
38	0.088350106	0.118082579
40	0.087009457	0.116786058
42	0.090276337	0.114759636
44	0.087612216	0.106564478
46	0.081124896	0.115053788
48	0.085472284	0.108325619
50	0.091583973	0.106902817
52	0.084651182	0.109758777
54	0.09395763	0.105208888
56	0.094935335	0.10991654
58	0.091305357	0.106227923
60	0.089515732	0.102030237
62	0.105699115	0.102786319
64	0.108520728	0.099828413
66	0.083646068	0.093414478
68	0.090620394	0.09736398
70	0.090169535	0.100323498
72	0.104800344	0.108090162
74	0.105019308	0.100520873
76	0.08266998	0.097670605

78	0.081432832	0.090647249
80	0.078018528	0.099781088
82	0.094112599	0.098722838
84	0.094279093	0.090689204
86	0.091746977	0.113551788
88	0.097671568	0.086862636
90	0.099526806	0.086393084
92	0.07636361	0.089969627
94	0.083790312	0.096383782
96	0.081816169	0.089555744
98	0.079272888	0.094985159
100	0.104893329	0.09750446
150	0.085002216	0.098511639
200	0.091091786	0.082102492
250	0.082605022	0.091424834
300	0.07588406	0.088323532
350	0.072220735	0.087057681
400	0.075645462	0.08687107
450	0.097525982	0.104273715
500	0.071986377	0.086109729
600	0.09021143	0.076351046
700	0.084034261	0.081856292
800	0.075735933	0.075965901
900	0.081818557	0.090287901
1000	0.086410697	0.08259724
Full	0.07571072	0.082683398

Table S3. Hyperparameter optimization results of descriptor-based DNN and fingerprint-based DNN.

Feature	Mean squared error (MSE)	Learning rate	Number of layers	Number of nodes	Optimizer	Dropout rate	L2 regularization rate	Batch size
Descriptor	0.072	0.100	3	295	sgd	0.3	0.00001	64
Descriptor	0.077	0.100	1	298	sgd	0.3	0.00001	64
Descriptor	0.082	0.100	1	246	sgd	0.3	0.00001	64
Descriptor	0.082	0.100	6	128	sgd	0.5	0.00001	64
Descriptor	0.085	0.100	6	128	sgd	0.5	0.00001	128
Descriptor	0.086	0.100	1	295	sgd	0.3	0.00001	64
Descriptor	0.092	0.100	1	324	sgd	0.3	0.00001	64
Descriptor	0.092	0.062	5	362	sgd	0.4	0.00001	64
Descriptor	0.093	0.100	1	297	sgd	0.3	0.00001	64
Descriptor	0.094	0.100	6	294	sgd	0.3	0.00001	64
Descriptor	0.096	0.100	1	295	sgd	0.3	1.00E-05	64
Descriptor	0.098	0.100	1	295	sgd	0.3	0.00001	64
Descriptor	0.101	0.100	6	128	sgd	0.4	0.00001	64
Descriptor	0.101	0.100	1	512	sgd	0.5	0.00001	64
Descriptor	0.102	0.100	1	294	sgd	0.3	1.37805E-05	64
Descriptor	0.106	0.100	1	396	sgd	0.3	2.11965E-05	128
Descriptor	0.108	0.001	3	159	sgd	0.4	0.00001	128
Descriptor	0.114	0.100	2	424	sgd	0.3	0.00001	128
Descriptor	0.120	0.100	6	437	sgd	0.5	0.00001	128
Descriptor	0.120	0.100	1	380	sgd	0.3	1.49E-05	256
Descriptor	0.120	0.019	6	271	sgd	0.4	0.00001	128
Descriptor	0.130	0.100	6	373	sgd	0.5	1.53584E-05	128
Descriptor	0.142	0.100	1	254	sgd	0.4	6.68547E-05	256

Descriptor	0.160	0.001	6	306	sgd	0.3	1.27745E-05	128
Descriptor	0.172	0.001	5	362	sgd	0.3	2.66421E-05	64
Descriptor	0.174	0.000	2	148	adam	0.4	7.77294E-05	256
Descriptor	0.174	0.000	1	128	adam	0.5	0.000221335	128
Descriptor	0.183	0.001	1	312	adam	0.5	0.01	128
Descriptor	0.193	0.001	1	128	adam	0.5	0.000220413	256
Descriptor	0.201	0.100	6	128	adam	0.4	0.01	64
Descriptor	0.214	0.001	1	506	adam	0.5	0.000798096	64
Descriptor	0.215	0.066	6	128	sgd	0.3	0.000121226	128
Descriptor	0.245	0.001	6	128	adam	0.5	0.01	64
Descriptor	0.254	0.000	1	128	adam	0.5	7.7638E-05	256
Descriptor	0.254	0.100	1	128	adam	0.5	0.00001	128
Descriptor	0.266	0.000	3	128	adam	0.3	1.00E-05	64
Descriptor	0.271	0.099	3	452	adam	0.5	0.0032384	128
Descriptor	0.281	0.069	2	494	adam	0.4	0.01	256
Descriptor	0.285	0.003	1	128	sgd	0.3	0.00001	256
Descriptor	0.297	0.002	1	207	adam	0.4	7.07E-04	256
Descriptor	0.298	0.100	6	128	adam	0.4	0.000937362	256
Descriptor	0.300	0.100	1	128	adam	0.5	3.80E-03	128
Descriptor	0.300	0.000	6	512	sgd	0.4	1.00E-05	256
Descriptor	0.309	0.100	1	128	adam	0.5	4.72E-04	64
Descriptor	0.312	0.100	6	386	sgd	0.4	1.50E-05	128
Descriptor	0.322	0.070	1	139	sgd	0.5	6.38E-05	256
Descriptor	0.327	0.000	6	128	sgd	0.5	0.00001	128
Descriptor	0.327	0.100	2	128	sgd	0.5	1.00E-05	128
Descriptor	0.329	0.070	1	186	sgd	0.4	1.00E-05	256

Descriptor	0.340	0.000	1	128	adam	0.4	0.000181384	64
Descriptor	0.349	0.001	1	128	adam	0.4	0.01	256
Descriptor	0.363	0.010	4	128	sgd	0.4	0.00001	128
Descriptor	0.395	0.000	6	512	adam	0.3	0.01	64
Descriptor	0.408	0.100	1	128	sgd	0.3	0.01	256
Descriptor	0.412	0.026	4	161	adam	0.4	8.85E-05	256
Descriptor	0.454	0.091	2	130	adam	0.3	8.17E-04	64
Descriptor	0.458	0.001	6	128	adam	0.4	0.01	64
Descriptor	0.476	0.000	6	193	sgd	0.3	0.00001	64
Descriptor	0.489	0.100	6	128	adam	0.3	0.00001	64
Descriptor	0.492	0.100	6	128	sgd	0.3	0.00015298	128
Descriptor	0.506	0.000	6	512	sgd	0.4	2.65442E-05	256
Descriptor	0.519	0.008	5	512	adam	0.4	8.46075E-05	128
Descriptor	0.520	0.100	6	128	sgd	0.3	0.01	128
Descriptor	0.559	0.001	3	338	adam	0.3	0.006816979	256
Descriptor	0.570	0.100	6	512	adam	0.5	0.01	128
Descriptor	0.577	0.100	6	512	sgd	0.5	1.00E-05	128
Descriptor	0.584	0.000	1	486	sgd	0.3	1.37185E-05	128
Descriptor	0.594	0.000	1	128	adam	0.4	0.000560648	256
Descriptor	0.602	0.001	4	403	adam	0.4	1.21E-03	64
Descriptor	0.621	0.000	6	128	adam	0.5	3.95E-03	64
Descriptor	0.631	0.016	1	128	adam	0.5	0.01	64
Descriptor	0.680	0.000	1	215	adam	0.3	5.12834E-05	256
Descriptor	0.744	0.100	3	128	sgd	0.3	1.00E-02	256
Descriptor	0.806	0.100	6	128	sgd	0.3	0.01	256
Descriptor	0.811	0.100	6	128	sgd	0.3	1.00E-02	256

Descriptor	0.897	0.000	1	128	adam	0.5	1.68E-03	256
Descriptor	0.939	0.100	6	128	sgd	0.4	0.000595982	128
Descriptor	1.014	0.000	1	512	adam	0.5	5.33698E-05	256
Descriptor	1.030	0.000	1	489	adam	0.3	1.00E-05	256
Descriptor	1.091	0.100	1	128	sgd	0.3	0.01	256
Descriptor	1.092	0.100	6	128	sgd	0.3	0.01	256
Descriptor	1.212	0.100	1	128	sgd	0.3	0.01	256
Descriptor	1.867	0.100	6	512	sgd	0.3	0.00015634	256
Descriptor	1.964	0.014	6	428	adam	0.4	0.006281789	256
Descriptor	1.979	0.067	3	433	sgd	0.4	0.008170944	128
Descriptor	2.202	0.000	1	396	adam	0.4	0.00001	64
Descriptor	2.301	0.011	3	465	sgd	0.3	1.21E-03	64
Descriptor	2.467	0.100	4	423	adam	0.3	0.000598048	256
Descriptor	2.597	0.012	2	462	sgd	0.5	0.002259779	64
Descriptor	2.861	0.010	3	512	sgd	0.5	0.01	128
Descriptor	2.952	0.000	1	128	adam	0.4	0.008893893	128
Descriptor	3.241	0.000	1	128	adam	0.4	1.00E-02	256
Descriptor	3.640	0.000	2	450	adam	0.3	0.002119927	64
Descriptor	3.659	0.000	1	128	sgd	0.3	0.01	256
Descriptor	3.691	0.000	1	128	sgd	0.3	0.01	128
Descriptor	5.067	0.000	6	391	sgd	0.5	1.68E-03	256
Descriptor	10.136	0.000	6	128	adam	0.4	0.01	64
Descriptor	11.178	0.000	1	512	adam	0.4	0.01	64
Descriptor	15.017	0.012	4	128	adam	0.4	1.00E-05	256
Descriptor	120.036	0.100	6	512	adam	0.3	0.000281907	128
Fingerprint	0.076	0.100	1	128	sgd	0.3	1.00E-05	64

Fingerprint	0.082	0.100	1	128	sgd	0.3	0.00001	64
Fingerprint	0.083	0.100	1	128	sgd	0.3	0.00001	64
Fingerprint	0.084	0.100	1	128	sgd	0.3	0.00001	64
Fingerprint	0.087	0.007	6	128	adam	0.5	0.00001	64
Fingerprint	0.088	0.100	1	128	sgd	0.3	0.00001	64
Fingerprint	0.091	0.100	1	128	sgd	0.3	1.00E-05	64
Fingerprint	0.091	0.100	1	171	sgd	0.3	0.00001	64
Fingerprint	0.093	0.100	6	128	sgd	0.4	0.00001	64
Fingerprint	0.094	0.100	1	128	sgd	0.3	0.00001	64
Fingerprint	0.096	0.100	1	128	sgd	0.3	1.00E-05	64
Fingerprint	0.104	0.002	4	147	adam	0.4	2.96E-05	256
Fingerprint	0.105	0.100	1	512	sgd	0.3	0.00001	64
Fingerprint	0.107	0.002	1	332	adam	0.4	3.53963E-05	256
Fingerprint	0.118	0.004	1	257	adam	0.4	0.01	128
Fingerprint	0.131	0.002	1	217	adam	0.4	0.003028869	128
Fingerprint	0.131	0.000	1	128	sgd	0.5	3.32977E-05	256
Fingerprint	0.138	0.034	6	512	sgd	0.4	1.00E-05	64
Fingerprint	0.156	0.015	6	422	sgd	0.4	2.12591E-05	64
Fingerprint	0.166	0.000	1	464	adam	0.5	1.15E-03	128
Fingerprint	0.168	0.001	6	169	sgd	0.3	1.00E-05	128
Fingerprint	0.176	0.004	2	218	adam	0.4	1.34E-04	256
Fingerprint	0.177	0.100	2	158	adam	0.4	0.00034099	64
Fingerprint	0.194	0.000	6	169	adam	0.4	9.41E-03	64
Fingerprint	0.198	0.000	1	128	adam	0.5	1.00E-05	128
Fingerprint	0.207	0.100	1	128	adam	0.3	0.00001	64
Fingerprint	0.211	0.002	3	443	adam	0.4	8.51E-04	256

Fingerprint	0.220	0.000	1	512	sgd	0.5	9.10782E-05	256
Fingerprint	0.223	0.001	6	209	adam	0.3	1.67E-03	64
Fingerprint	0.227	0.002	4	221	adam	0.4	3.24E-03	256
Fingerprint	0.229	0.072	5	474	sgd	0.5	4.60818E-05	64
Fingerprint	0.240	0.002	6	487	adam	0.5	4.00E-05	128
Fingerprint	0.242	0.003	5	315	adam	0.5	9.17E-05	64
Fingerprint	0.250	0.000	6	128	sgd	0.5	0.00001	256
Fingerprint	0.257	0.000	4	128	sgd	0.5	0.00001	256
Fingerprint	0.265	0.100	1	128	sgd	0.3	0.01	128
Fingerprint	0.266	0.000	6	128	sgd	0.4	1.00E-05	256
Fingerprint	0.272	0.006	1	128	sgd	0.3	4.16718E-05	64
Fingerprint	0.276	0.048	1	339	adam	0.4	1.00E-02	128
Fingerprint	0.289	0.000	6	128	sgd	0.3	1.00E-05	128
Fingerprint	0.303	0.100	6	512	adam	0.3	0.00513554	64
Fingerprint	0.309	0.000	2	510	adam	0.5	3.73E-03	256
Fingerprint	0.313	0.000	6	128	sgd	0.4	5.43571E-05	256
Fingerprint	0.320	0.002	1	351	sgd	0.4	6.31E-05	128
Fingerprint	0.324	0.084	2	201	sgd	0.3	1.00E-05	256
Fingerprint	0.331	0.000	1	128	sgd	0.3	1.00E-05	256
Fingerprint	0.332	0.100	6	128	sgd	0.3	1.00E-05	128
Fingerprint	0.332	0.000	5	128	sgd	0.5	1.00E-05	256
Fingerprint	0.334	0.100	1	512	adam	0.4	0.003812486	64
Fingerprint	0.336	0.009	1	391	sgd	0.3	0.00001	256
Fingerprint	0.343	0.100	6	128	sgd	0.4	0.01	64
Fingerprint	0.351	0.012	1	205	sgd	0.3	5.87658E-05	256
Fingerprint	0.369	0.100	4	128	sgd	0.3	0.00001	128

Fingerprint	0.385	0.100	6	128	sgd	0.4	4.83102E-05	128
Fingerprint	0.386	0.100	4	128	sgd	0.5	0.00001	256
Fingerprint	0.386	0.100	6	128	sgd	0.5	0.00001	256
Fingerprint	0.396	0.058	5	140	adam	0.5	1.89668E-05	256
Fingerprint	0.402	0.100	2	188	adam	0.5	0.00001	64
Fingerprint	0.417	0.001	6	128	sgd	0.3	0.000260928	128
Fingerprint	0.434	0.100	3	201	adam	0.5	0.00001	64
Fingerprint	0.450	0.100	6	512	adam	0.3	0.001059718	64
Fingerprint	0.453	0.000	6	512	sgd	0.3	0.00001	128
Fingerprint	0.455	0.000	6	128	sgd	0.4	0.000221428	64
Fingerprint	0.465	0.004	6	469	adam	0.5	0.000131465	128
Fingerprint	0.479	0.100	1	310	sgd	0.3	0.00053043	64
Fingerprint	0.499	0.004	4	376	adam	0.4	2.87318E-05	256
Fingerprint	0.503	0.001	3	459	adam	0.4	0.00079776	256
Fingerprint	0.550	0.002	6	128	sgd	0.4	0.000415649	128
Fingerprint	0.555	0.000	1	128	sgd	0.5	0.00001	128
Fingerprint	0.570	0.100	3	128	adam	0.4	0.000149946	128
Fingerprint	0.611	0.000	4	415	adam	0.3	1.58773E-05	128
Fingerprint	0.614	0.100	6	128	sgd	0.5	0.01	256
Fingerprint	0.622	0.100	1	512	sgd	0.5	0.01	128
Fingerprint	0.633	0.000	1	128	adam	0.4	0.00039334	128
Fingerprint	0.668	0.000	4	512	sgd	0.5	0.00001	64
Fingerprint	0.775	0.001	6	512	adam	0.5	0.000201703	128
Fingerprint	0.806	0.000	1	128	adam	0.5	0.000378265	128
Fingerprint	0.884	0.000	1	512	sgd	0.3	0.000311604	64
Fingerprint	0.982	0.000	2	512	adam	0.3	0.00001	128

Fingerprint	1.078	0.005	2	128	sgd	0.3	0.001695399	128
Fingerprint	1.170	0.000	3	450	adam	0.3	0.000152263	128
Fingerprint	1.183	0.012	1	297	sgd	0.3	0.001216333	256
Fingerprint	1.225	0.000	2	477	sgd	0.3	0.000579032	256
Fingerprint	1.293	0.000	5	510	adam	0.3	0.00001	128
Fingerprint	1.492	0.023	1	128	sgd	0.3	0.003429297	256
Fingerprint	2.085	0.000	1	498	adam	0.4	0.00001	64
Fingerprint	2.414	0.081	1	128	adam	0.4	0.000439249	128
Fingerprint	2.746	0.000	6	145	adam	0.3	0.00170569	64
Fingerprint	2.875	0.000	4	512	adam	0.5	0.00001	64
Fingerprint	3.172	0.010	3	512	sgd	0.5	0.01	128
Fingerprint	3.867	0.000	1	361	sgd	0.4	0.004162825	64
Fingerprint	7.073	0.014	1	285	sgd	0.5	0.01	256
Fingerprint	9.521	0.009	6	377	sgd	0.4	0.0040638	256
Fingerprint	11.878	0.000	3	400	sgd	0.4	0.007164412	256
Fingerprint	13.966	0.001	6	405	sgd	0.5	0.005721148	64
Fingerprint	22.041	0.000	4	413	sgd	0.3	0.01	128
Fingerprint	31.705	0.017	4	284	adam	0.3	0.000273236	256
Fingerprint	35.055	0.100	2	405	adam	0.4	0.000343458	128
Fingerprint	72.855	0.100	6	512	adam	0.4	3.1144E-05	128
Fingerprint	91.711	0.100	5	336	adam	0.4	0.00001	256

Table S4. Optimal hyperparameters used in this study.

Model	Mean squared error (MSE)	Learning rate	Number of layers	Number of nodes	Optimizer	Dropout rate	L2 regularization rate	Batch size
Descriptor-based DNN	0.072	0.100	3	295	sgd	0.3	0.00001	64
Fingerprint-based DNN	0.076	0.100	1	128	sgd	0.3	1.00E-05	64
